# Supplementary material for: SnRK1-triggered switch of bZIP63 dimerization mediates the low-energy response in plants
Source: eLife. 2015 Aug 11;4:e05828. doi: 10.7554/eLife.05828 (PMC4558565; doi:10.7554/eLife.05828)
Supplement: Figure 4—source data 1. — DOI: http://dx.doi.org/10.7554/eLife.05828.016 [file elife05828s003.docx]

**Figure 4 – source data 2. Overview over the kinases identified by LC-MS/MS after affinity purification with bZIP63**

|  |  |  |  |  |  |  |  |
| --- | --- | --- | --- | --- | --- | --- | --- |
| **Kinase family** | **found in** | | **Kinase subuni^1^** | | | | |
|  | **IP-MS/MS^2^** | | **Protein name^4^** | | **AGI^5^** | **MW** | **Subcellular localization** |
|  | **Yes/No** | **# of finds^3^** |  |  |  | **(all s.f.)^6^** | **shown by GFP fusion^7^** |
| **SnRK1** | **Yes** | **9** | **AKIN10 / SnRK1.1** | **SNF1 Kinase homolog 10, SNF1-related protein kinase 1.1** | At3g01090 | 58/61/58 | **N**[1,2], C[1,2,3], CP[3] |
|  | **Yes** | **7/8*** | **AKIN11 / SnRK1.2** | **SNF1 Kinase homolog 11, SNF1-related protein kinase 1.2** | At3g29160 | 59/59/41 | **N**[1,2], C[1,2,3], CP[3] |
|  | Yes | 0/1* | AKIN12 / SnRK1.3 | SNF1 Kinase homolog 12, SNF1-related protein kinase 1.3 | At5g39440 | 57 | ? |
|  | No |  | AKINb1 | SnRK1 kinase regulatory subunit beta-1 | At5g21170 | 31/35 | PM[4], **N**[4] |
|  | Yes | 1 | AKINb2 | SnRK1 kinase regulatory subunit beta-2 | At4g16360 | 29/29/29 | PM[4], C[4,5], **N**[5] |
|  | No |  | AKINb3 | SnRK1 kinase regulatory subunit beta-3 | At2g28060 | 13 | **N**[5], C[5] |
|  | **Yes** | **9** | **SNF4** | **Sucrose Nonfermenting 4** | At1g09020 | 53/43 | **N**[2,5], C[2,5] |
| **CDPKs** | **Yes** | **3** | **CPK3 / CDPK6** | **Calcium-dependent protein kinase 3** | At4g23650 | 59 | C[6,7,8], **N**[6,7,8], PM[7], VM[7] |
|  | Yes | 1 | CPK5 | Calcium-dependent protein kinase 5 | At4g35310 | 63 | C[9], **N**[9] |
|  | Yes? | 0/2* | CPK4 | Calcium-dependent protein kinase 4 | At4g09570 | 56 | C[6,9,10], **N**[9,10] |
|  | Yes? | 0/2* | CPK11 / CDPK2 | Calcium-dependent protein kinase 11 | At1g35670 | 56 | C[9,10,11], **N**[9,10,11] |
|  | Yes | 1 | CPK9 | Calcium-dependent protein kinase 9 | At3g20410 | 60 | PM[6,12,13,14] |
| **CKII** | **Yes** | **7/8*** | **CKA1** | **Casein Kinase II alpha chain 1** | At5g67380 | 48/44 | **N**[15] |
|  | **Yes** | **9/10*** | **CKA2** | **Casein Kinase II alpha chain 2** | At3g50000 | 47 | **N**[15] |
|  | No |  | CKA3 | Casein Kinase II alpha chain 3 | At2g23080 | 39/36 | **N**[15] |
|  | Yes | 7 | CKAcp | Casein Kinase II chloroplastidic alpha chain | At2g23070 | 50 | CP[1,15] |
|  | **Yes** | **7** | **CKB1** | **Casein Kinase II beta chain 1** | At5g47080 | 32/29/28/32 | **N**[15,16], C[15,16?] |
|  | No |  | CKB2 | Casein Kinase II beta chain 2 | At4g17640 | 32/31 | **N**[15,16] |
|  | No |  | CKB3 | Casein Kinase II beta chain 3 | At3g60250 | 31/31 | **N**[15], C[15] |
|  | No |  | CKB4 | Casein Kinase II beta chain 4 | At2g44680 | 32/32 | C[15,17], **N**[17] |
| **other** | **Yes** | **2** | **CKL2** | **Casein kinase I-like protein 2** | At1g72710 | 52 | **N**[18], C[18] |
|  | Yes | 1 | MPK16 | Mitogen-activated protein kinase 16 | At5g19010 | 56 | ? |
|  | Yes? | 0/1* | CDKC2 | Cyclin-dependent kinase C2 | At5g64960 | 57/51 | **N**[19,20] |
|  | Yes? | 0/1* | CDKC1 | Cyclin-dependent kinase C1 | At5g10270 | 57 | **N**[21] |
|  | Yes | 1 | CRK9, EP1 | Cysteine-Rich RLK (Receptor-Like Protein Kinase) 9 | At4g23170 | 30 | ? |
|  |  |  |  |  |  |  |  |

1 List of all kinase complex subunits identified in the LC-MS/MS approach to find bZIP63 upstream kinases. For SnRK1 and CKII, the remaining, but not identified kinase subunits, are also included. Kinase subunits which were identified with proteotypic peptides in more than one sample, have approximately the expected size, and don’t have a contradicting subcellular localization were considered high confidence candidates and are shown in black. Low confidence kinases subunits and kinases subunits not found are shown in grey and reasons for exclusion from the high confidence list are underlined.
2 Found by immunoprecipitation followed by tandem mass spectrometry?
3 Number of samples in which the protein was found (* including samples without proteotypic peptide for this kinase subunit).
4 Short and long name of the kinase complex subunits.
5 gene identifier according to TAIR (www.arabidopsis.org).
6 Molecular weight of all splicing forms (s.f.).
7 Published subcellular localization of the protein as shown by GFP fusion: N (nucleus), C (cytoplasm), CP (chloroplast), PM (plasma membrane), VM (vacuolar membrane). References: [1] Bayer et al., 2012; [2] Bitrian et al., 2011; [3] Fragoso et al., 2009; [4] Pierre et al., 2007; [5] Gissot et al., 2006; [6] Dammann et al., 2003; [7] Mehlmer et al., 2010; [8] Berendzen et al., 2012; [9] Boudsocq et al., 2010; [10] Zhu et al., 2007; [11] Rodriguez Milla et al., 2006; [12] Benetka et al., 2008; [13] Dong et al., 2008; [14] Padmanaban et al., 2007; [15] Salinas et al., 2006; [16] Park et al., 2008; [17] Perales et al., 2006; [18] Lee et al., 2005; [19] Koroleva et al., 2005; [20] Kitsios et al., 2008; [21] Boruc et al., 2010

**References**

Bayer RG, Stael S, Rocha AG, Mair A, Vothknecht UC, Teige M. 2012. Chloroplast-localized protein kinases: a step forward towards a complete inventory. *Journal of Experimental Botany* **63**: 1713-23. doi: 10.1093/jxb/err377

Benetka W, Mehlmer N, Maurer-Stroh S, Sammer M, Koranda M, Neumuller R, Betschinger J, Knoblich JA, Teige M, Eisenhaber F. 2008. Experimental testing of predicted myristoylation targets involved in asymmetric cell division and calcium-dependent signalling. *Cell Cycle* **7**: 3709-19. doi: 10.4161/cc.7.23.7176

Berendzen KW, Bohmer M, Wallmeroth N, Peter S, Vesic M, Zhou Y, Tiesler FK, Schleifenbaum F, Harter K. 2012. Screening for in planta protein-protein interactions combining bimolecular fluorescence complementation with flow cytometry. *Plant Methods* **8**: 25. doi: 10.1186/1746-4811-8-25

Bitrian M, Roodbarkelari F, Horvath M, Koncz C. 2011. BAC-recombineering for studying plant gene regulation: developmental control and cellular localization of SnRK1 kinase subunits. *The Plant Journal* **65**: 829-42. doi: 10.1111/j.1365-313X.2010.04462.x

Boruc J, Mylle E, Duda M, De Clercq R, Rombauts S, Geelen D, Hilson P, Inze D, Van Damme D,.Russinova E. 2010. Systematic localization of the Arabidopsis core cell cycle proteins reveals novel cell division complexes. *Plant Physiology* **152**: 553-65. doi: 10.1104/pp.109.148643

Boudsocq M, Willmann MR, McCormack M, Lee H, Shan L, He P, Bush J, Cheng SH, Sheen J. 2010. Differential innate immune signalling via Ca(2+) sensor protein kinases. *Nature* **464**: 418-22. doi: 10.1038/nature08794

Dammann C, Ichida A, Hong B, Romanowsky SM, Hrabak EM, Harmon AC, Pickard BG, Harper JF. 2003. Subcellular targeting of nine calcium-dependent protein kinase isoforms from Arabidopsis. *Plant Physiology* **132**: 1840-48. doi: 10.1104/pp.103.020008

Dong CH, Rivarola M, Resnick JS, Maggin BD, Chang C. 2008. Subcellular co-localization of Arabidopsis RTE1 and ETR1 supports a regulatory role for RTE1 in ETR1 ethylene signaling. *The Plant Journal* **53**: 275-86. doi: 10.1111/j.1365-313X.2007.03339.x

Fragoso S, Espindola L, Paez-Valencia J, Gamboa A, Camacho Y, Martinez-Barajas E, Coello P. 2009. SnRK1 isoforms AKIN10 and AKIN11 are differentially regulated in Arabidopsis plants under phosphate starvation. *Plant Physiology* **149**: 1906-16. doi: 10.1104/pp.108.133298

Gissot L, Polge C, Jossier M, Girin T, Bouly JP, Kreis M, Thomas M. 2006. AKINbetagamma contributes to SnRK1 heterotrimeric complexes and interacts with two proteins implicated in plant pathogen resistance through its KIS/GBD sequence. *Plant Physiology* **142**: 931-44. doi: 10.1104/pp.106.087718

Kitsios G, Alexiou KG, Bush M, Shaw P, Doonan JH. 2008. A cyclin-dependent protein kinase, CDKC2, colocalizes with and modulates the distribution of spliceosomal components in Arabidopsis. *The Plant Journal* **54**: 220-35. doi: 10.1111/j.1365-313X.2008.03414.x

Koroleva OA, Tomlinson ML, Leader D, Shaw P, Doonan JH. 2005. High-throughput protein localization in Arabidopsis using Agrobacterium-mediated transient expression of GFP-ORF fusions. *The Plant Journal* **41**: 162-74. doi: 10.1111/j.1365-313X.2004.02281.x

Lee JY, Taoka K, Yoo BC, Ben-Nissan G, Kim DJ, Lucas WJ. 2005. Plasmodesmal-associated protein kinase in tobacco and Arabidopsis recognizes a subset of non-cell-autonomous proteins. *The Plant Cell* **17**: 2817-31. doi: 10.1105/tpc.105.034330

Mehlmer N, Wurzinger B, Stael S, Hofmann-Rodrigues D, Csaszar E, Pfister B, Bayer R, Teige M. 2010. The Ca(2+) -dependent protein kinase CPK3 is required for MAPK-independent salt-stress acclimation in Arabidopsis. *The Plant Journal* **63**: 484-98. doi: 10.1111/j.1365-313X.2010.04257.x

Padmanaban S, Chanroj S, Kwak JM, Li X, Ward JM, Sze H. 2007. Participation of endomembrane cation/H+ exchanger AtCHX20 in osmoregulation of guard cells. *Plant Physiology* **144**: 82-93. doi: 10.1104/pp.106.092155

Park HJ, Ding L, Dai M, Lin R, Wang H. 2008. Multisite phosphorylation of Arabidopsis HFR1 by casein kinase II and a plausible role in regulating its degradation rate. *The Journal of biological chemistry* **283**: 23264-73. doi: 10.1074/jbc.M801720200

Perales M, Portoles S, Mas P. 2006. The proteasome-dependent degradation of CKB4 is regulated by the Arabidopsis biological clock. *The Plant Journal* **46**: 849-60. doi: 10.1111/j.1365-313X.2006.02744.x

Pierre M, Traverso JA, Boisson B, Domenichini S, Bouchez D, Giglione C, Meinnel T. 2007. N-myristoylation regulates the SnRK1 pathway in Arabidopsis. *The Plant Cell* **19**: 2804-21. doi: 10.1105/tpc.107.051870

Rodriguez Milla MA, Uno Y, Chang IF, Townsend J, Maher EA, Quilici D, Cushman JC. 2006. A novel yeast two-hybrid approach to identify CDPK substrates: characterization of the interaction between AtCPK11 and AtDi19, a nuclear zinc finger protein. *FEBS Letters* **580**: 904-11. doi: 10.1016/j.febslet.2006.01.013

Salinas P, Fuentes D, Vidal E, Jordana X, Echeverria M, Holuigue L. 2006. An extensive survey of CK2 alpha and beta subunits in Arabidopsis: multiple isoforms exhibit differential subcellular localization. *Plant Cell Physiology* **47**: 1295-1308. doi: 10.1093/pcp/pcj100

Zhu SY, Yu XC, Wang XJ, Zhao R, Li Y, Fan RC, Shang Y, Du SY, Wang XF, Wu FQ, Xu YH, Zhang XY, Zhang DP. 2007. Two calcium-dependent protein kinases, CPK4 and CPK11, regulate abscisic acid signal transduction in Arabidopsis. *The Plant Cell* **19**: 3019-36. doi: 10.1105/tpc.107.050666
